# Green Synthesis of Euphorbia tirucalli-Mediated Titanium Dioxide Nanoparticles Against Wound Pathogens

**DOI:** 10.7759/cureus.53939

**Published:** 2024-02-09

**Authors:** Haritha Natarajan Sankar, Rajeshkumar Shanmugam, Jayasree Anandan

**Affiliations:** 1 Nanobiomedicine Lab, Centre for Global Health Research, Saveetha Medical College and Hospitals, Saveetha Institute of Medical and Technical Sciences, Chennai, IND; 2 Nanobiomedicine Lab, Centre for Global Health Research, Saveetha Medical College and Hospital, Saveetha Institute of Medical and Technical Sciences, Chennai, IND

**Keywords:** antimicrobial agent, wound infection, wound pathogens, euphorbia tirucalli, titanium dioxide nanoparticle

## Abstract

Background

Wound infections caused by pathogens present a considerable global health challenge, resulting in extended healing durations, elevated healthcare expenses, and potential fatalities. Conventional approaches to managing wound pathogens have limitations such as antibiotic resistance, toxicity and allergic reactions. Consequently, there is a rising interest in exploring alternative strategies for preventing and treating wound infections. Titanium dioxide nanoparticles (TiO_2_NPs) have gained attention for their potential in wound healing, attributed to their distinctive properties, including antimicrobial and anti-inflammatory capabilities.

Methods

TiO_2_NPs synthesized through *Euphorbia tirucalli* were examined for their antibacterial potential against wound pathogens, using the Kirby-Bauer agar-well diffusion method and time-kill curve assay. Furthermore, the cytotoxic effect of the synthesized nanoparticles was evaluated through a brine shrimp lethality assay.

Results

Green-synthesized TiO2NPs demonstrated potent antimicrobial activity against tested wound pathogens, displaying a zone of inhibition against *Pseudomonas aeruginosa* (11 mm) and *Escherichia coli* (10 mm) at the highest concentration of 100 μg/mL. In the time-kill curve assay, the prepared TiO_2_NPs showed significant bactericidal activity against *Pseudomonas aeruginosa* followed by *Escherichia coli*. In the brine shrimp lethality assay, at the lowest concentration of 5 μg/mL of the prepared nanoparticles, 100% of the nauplii remained alive after 48 hours.

Conclusion

The results indicate that TiO_2_NPs synthesized using *Euphorbia tirucalli* extract exhibit potent antimicrobial activity against the tested wound pathogens. Moreover, the prepared nanoparticles exhibit lower toxicity, suggesting their potential use as an alternative to commercially available synthetic drugs.

## Introduction

Wound healing is an intricate process with various stages, aiming to restore tissue integrity [1]. However, complications including wound infection and diabetes can hinder this process. Diabetes poses a significant challenge to wound healing, causing delays and complications. Elevated blood sugar levels in diabetes create an environment conducive to bacterial growth, increasing the risk of infections in wounds. Microbial invasion can lead to delayed healing, increased inflammation, and other complications [2]. Antimicrobial resistance (AMR) further complicates infection management, emphasizing the need for alternative approaches. Nanoparticles have unique physicochemical properties making them attractive candidates for biomedical applications [3].

Nanotechnology plays a crucial role in advancing wound healing by utilizing materials and structures at the nanoscale to improve the overall healing process [4]. Specifically, nanoparticles contribute significantly to innovative approaches that enhance wound healing. Nanoparticles, like silver and titanium nanoparticles, with inherent antimicrobial properties, can effectively prevent infections in wounds, creating a sterile environment conducive to healing [5]. Titanium dioxide nanoparticles (TiO_2_NPs), in particular, exhibit potential antimicrobial activity against various microorganisms and demonstrate excellent anti-inflammatory properties. Additionally, they promote cell proliferation and angiogenic effects and have been shown to reduce scarring [6].

*Euphorbia tirucalli*, commonly referred to as the “pencil cactus,” is a succulent plant recognized for its enormous medicinal properties [7]. This plant possesses excellent antimicrobial activity as it contains various bioactive compounds like terpenoids, flavonoids, and alkaloids. Extracts derived from *Euphorbia tirucalli* exhibit antibacterial efficacy against diverse bacterial strains [8]. The antimicrobial attributes of the plants hold the potential for safeguarding wounds against infections. *E. tirucalli* has a history of traditional use in certain cultures for wound healing and addressing various health concerns [9].

The core objective of this research is to investigate the antimicrobial activity of *Euphorbia tirucalli-*mediated titanium dioxide nanoparticles prepared with *Euphorbia tirucalli *against wound pathogens using agar-well diffusion method and time-kill curve assay, while also examining their cytotoxic effects using brine shrimp lethality assay. The study aims to explore the potential applications in health-related fields by investigating the antimicrobial efficacy of green-synthesized titanium dioxide nanoparticles. The results of this research offer promising prospects for developing alternatives to antimicrobials, with broader implications for sustainable and eco-friendly biomedical applications. 

## Materials and methods

Preparation of titanium dioxide nanoparticles

*Euphorbia tirucalli* stems were obtained from the Nano-Herbo garden located at Saveetha Dental College in Chennai. A 10 g *E. tirucalli* stem was precisely weighed and then crushed using a mortar and pestle. The crushed plant material was mixed with 100 mL of distilled water. The resulting extract underwent heating on a mantle at temperatures ranging from 50-60 degrees Celsius for duration of 15 minutes. Following this process, the extract was filtered through Whatman No.1 filter paper and stored for subsequent utilization. Titanium oxide (30 mM) was weighed and mixed with 50 mL of distilled water and the resulting titanium oxide solution was then combined with 50 mL of aqueous extract of *E. tirucalli *stem. This mixed solution underwent agitation in an orbital shaker for duration of 48 hours. The colour change of the solution mixture and UV-visible spectroscopy were taken for preliminary confirmation of the titanium dioxide nanoparticles. Following the 48-hour period, centrifugation was performed at 8000 RPM for the duration of 10 minutes. The resultant pellet was collected and retained for subsequent investigations.

Antimicrobial activity

The antimicrobial activity of the green-synthesized titanium dioxide nanoparticles was assessed using the agar-well diffusion technique against wound pathogens - *Pseudomonas aeruginosa* and *Escherichia coli*. The agar-well diffusion method was used to evaluate the antimicrobial activity of the prepared nanoparticles. Mueller-Hinton agar was used to cultivate the bacterial cultures and sterile petri plates were loaded with agar medium and left to solidify. The wells measuring 9 mm in diameter were established using sterilized polystyrene tips, and the test organisms were swabbed on the plates using sterile cotton swabs. Various concentrations of the nanoparticles (25, 50, and 100 μg/mL) were introduced into separate wells. The antibiotic amoxyrite was used as the standard. The plates were placed in an incubator at 37°C for 24 hours. After incubation, the zone of inhibition was measured in the plates. The assay was carried out following a methodology outlined in a previous study conducted by Ebenezer *et al* [10]. 

Time-kill curve assay

A 1 mL aliquot of the bacterial suspension (comprising *Pseudomonas aeruginosa* and *Escherichia coli*) was introduced into 9 mL of Mueller-Hinton broth, supplemented with TiO_2_NPs at concentrations of 25, 50, and 100 µg/mL. This resulted in a final microbial concentration of approximately 10^6^ CFU/mL. The mixture was subjected to incubation at 37°C with agitation at 200 RPM for various time intervals (0, 1, 2, 3, 4 and 5 h). Following that, the percentage of deceased cells was ascertained by measuring the absorbance at a wavelength of 600 nm at regular time intervals [11].

Cytotoxic effect 

The cytotoxic effect of the prepared TiO_2_NPs of various concentrations (5, 10, 20, 40, and 80 µg/mL) was examined using brine shrimp lethality assay (BSLA). The assay was carried out in accordance with the methodology outlined in a previous study conducted by Veerendrakumar *et al* [12].

## Results

Preliminary confirmation of titanium dioxide nanoparticles

**Figure 1:**
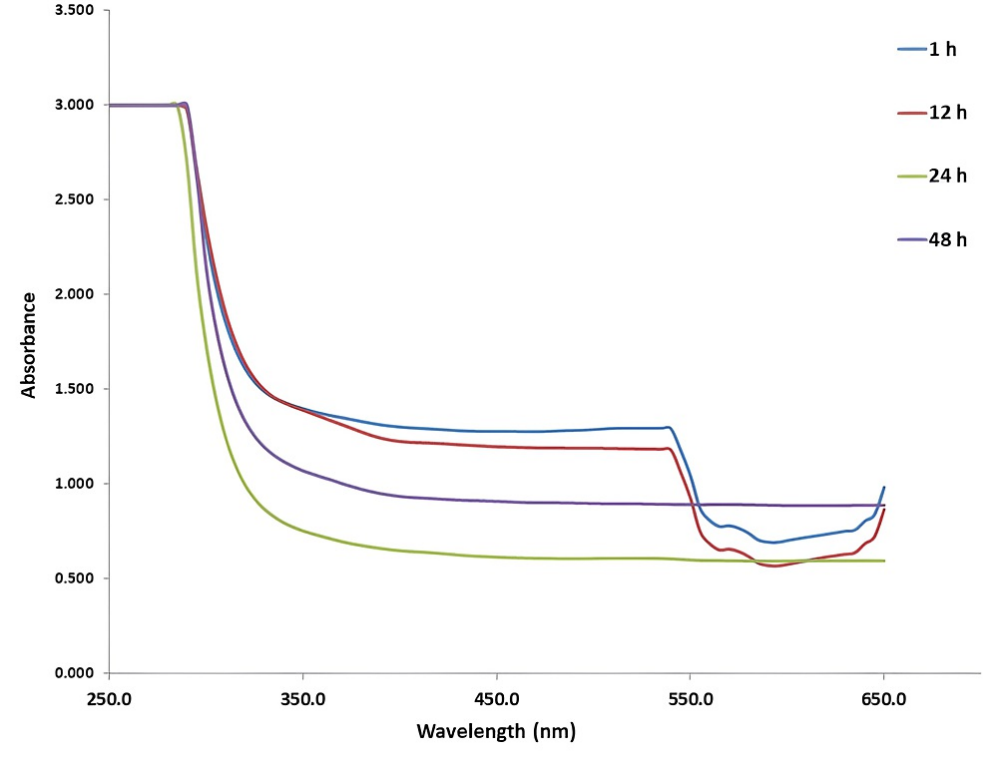
UV-visible spectra of E. tirucalli-mediated TiO2NPs E. tirucalli: *Euphorbia tirucalli*; TiO2NPs: titanium dioxide nanoparticles

Visual observation serves as the initial tool for analyzing nanoparticle synthesis. Following the addition of *E. tirucalli* extract to the titanium oxide precursor solution, the initial color was noted to be light pale white. The transition in color from pure whitish to a pale whitish shade was observed at the final stage, providing initial confirmation of the reducing and capping abilities of the *E. tirucalli* extract. Figure 1 depicts the UV-visible spectral analysis for *E. tirucalli*-mediated TiO_2_NPs, displaying a characteristic surface plasmon resonance (SPR) peak with maximum absorbance at 290 nm. This observation provides preliminary confirmation of the formation of TiO_2_NPs. 

Antimicrobial activity

**Figure 2:**
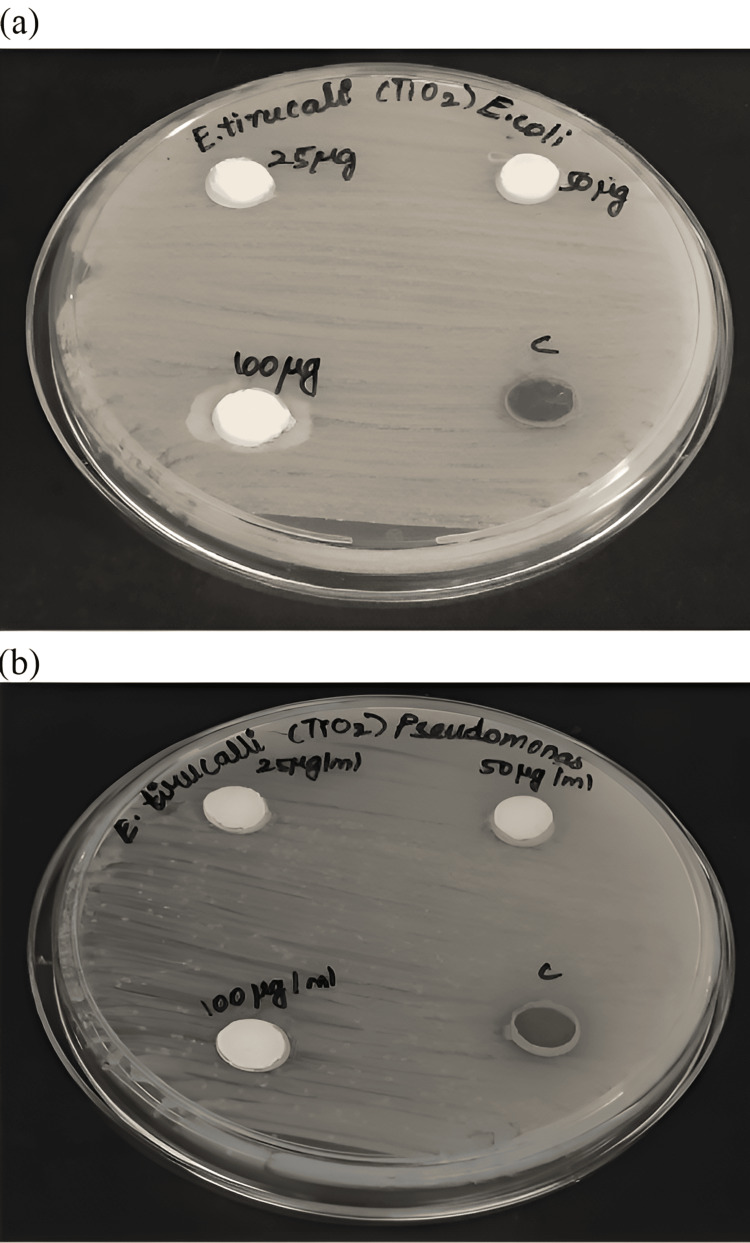
Picture representing the antimicrobial activity of TiO2NPs against wound pathogens (a) E. coli, (b) P. aeruginosa TiO2NPs: Titanium dioxide nanoparticles; E. coli: *Escherichia coli*; P. aeruginosa:  *Pseudomonas aeruginosa*

**Figure 3:**
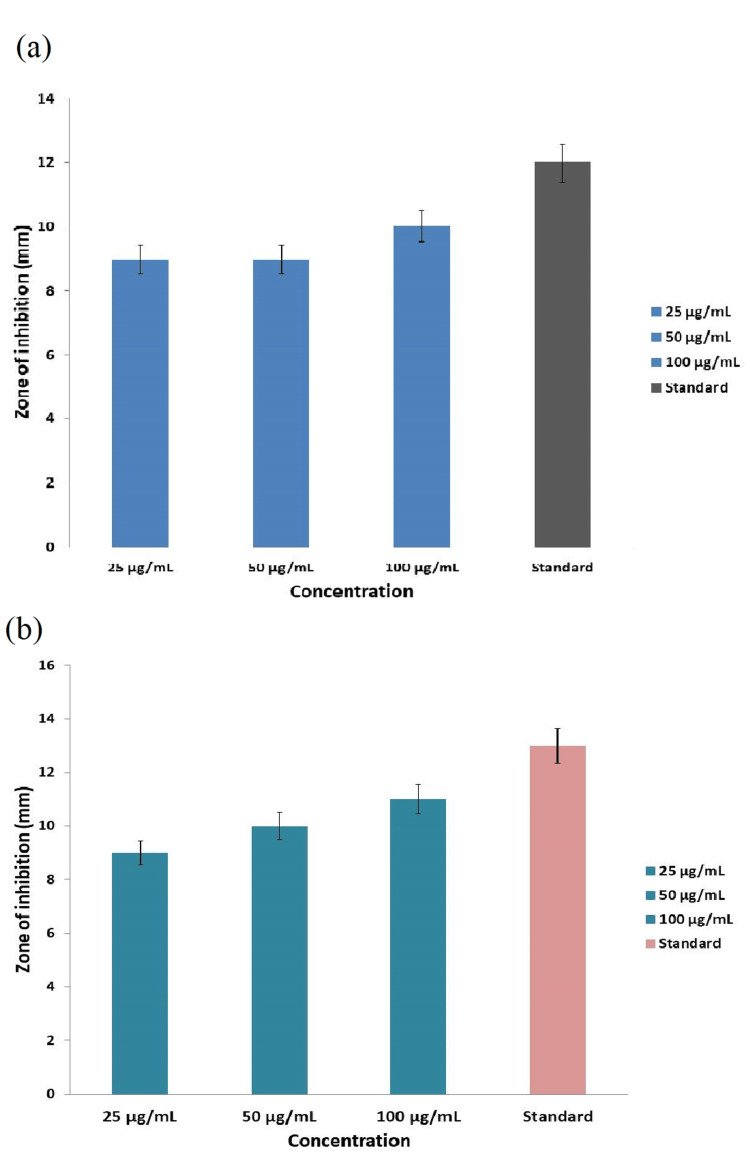
Graphs displaying antibacterial activity of E. tirucalli-mediated TiO2NPs against wound pathogens (a) E. coli, (b) P. aeruginosa E. tirucalli: *Euphorbia tirucalli*; TiO2NPs: titanium dioxide nanoparticles; E. coli: *Escherichia coli*; P. aeruginosa: *Pseudomonas aeruginosa*

The antimicrobial activity of titanium dioxide nanoparticles, synthesized using *E. tirucalli*, was assessed against wound pathogens (*Pseudomonas aeruginosa* and *Escherichia coli*). As depicted in Figure 2 and Figure 3, the antimicrobial effectiveness of the prepared nanoparticles showed a dose-dependent response on both the wound pathogens. However, in comparison to the standard amoxyrite, the green-synthesized TiO_2_NPs exhibited lower antimicrobial activity.

Time kill curve assay

**Figure 4:**
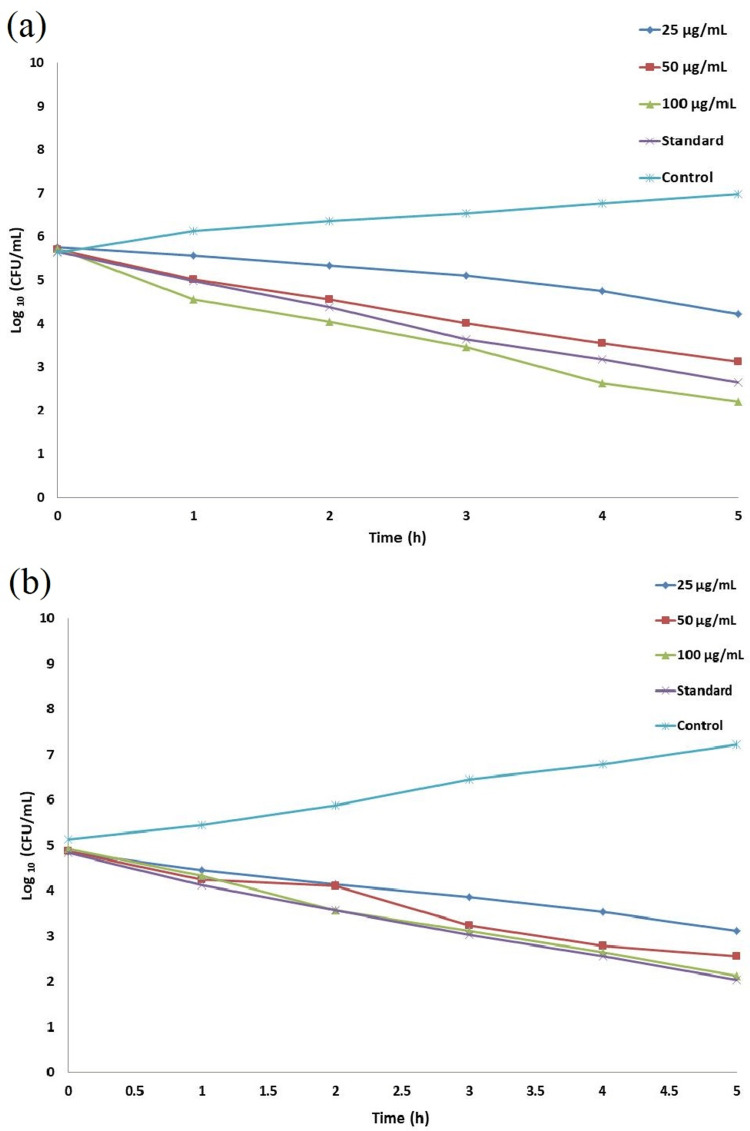
Time-kill curve assay of green synthesized TiO2NPs against wound pathogens (a) P. aeruginosa; (b) E. coli TiO2NPs: titanium dioxide nanoparticles; P. aeruginosa: *Pseudomonas aeruginosa*; E. coli: *Escherichia coli*

The results obtained from the time-kill curve assay highlight a reduction in viable cells for the tested wound pathogens attributed to the prepared TiO_2_NPs. In Figures 4a, 4b, the graphs representing the time-kill curve assay illustrate that the TiO_2_NPs display optimal bactericidal activity against *P. aeruginosa*. On *E. coli*, the synthesized NPs demonstrate mild bactericidal and bacteriostatic effects. The findings suggest that the bacteriostatic and bactericidal activities of the *E. tirucalli*-mediated TiO_2_NPs gradually manifest over time. Notably, at a concentration of 100 μg/mL, the produced nanoparticles demonstrate maximum bactericidal activity when compared to the standard amoxyrite against the tested organisms.

Cytotoxic effect

**Figure 5:**
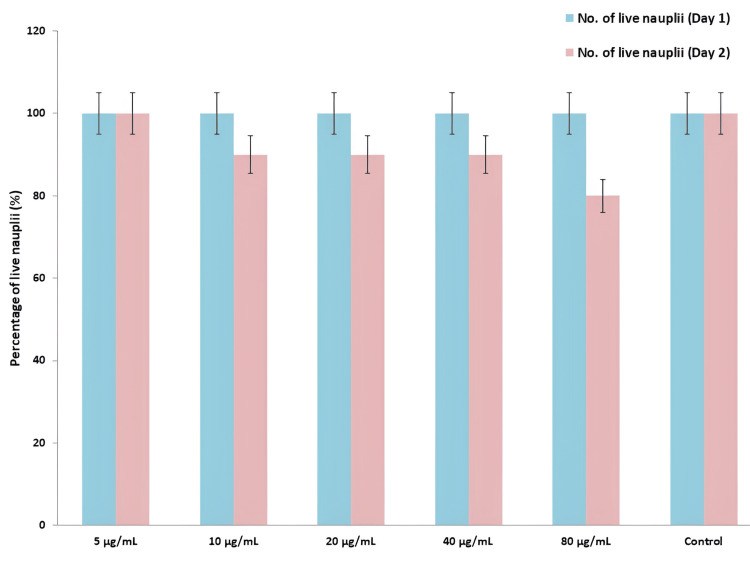
Cytotoxic effect of E. tirucalli-mediated TiO2NPs E. tirucalli: *Euphorbia tirucalli*; TiO2NPs: titanium dioxide nanoparticles

The cytotoxic effect of the TiO2NPs was evaluated using a BSLA, employing nauplii of *Artemia salina* as a model organism. As displayed in Figure 5, various concentrations (5-80 μL) of the TiO_2_NPs were tested against the brine shrimp nauplii. On the second day, the survival rate of brine shrimp nauplii remained consistent at the lowest concentration of 5 μg/mL, with a percentage of live nauplii ranging 100%. At a concentration of 10, 20, and 40 micrograms per mL, the percentage of live nauplii was 90%. At the highest concentration of 80 micrograms per mL, the percentage of live nauplii was observed to be 80%. All the nauplii in the control were present on both days 1 and 2.

## Discussion

The TiO_2_NPs-mediated through *E. tirucalli *exhibit potent antimicrobial activity against the wound pathogens, but while they display slightly lower antimicrobial activity compared to the standard amoxyrite, they demonstrate significant anti-microbial effects. In the brine shrimp lethality assay, the nanoparticles show less toxicity, with 100% viability in nauplii at the lowest concentration (5 μg/mL) and 80% at the highest concentration (80 μg/mL).

Scientists and researchers are actively investing in replacing traditional antibiotics to develop alternative therapeutic agents in response to the challenge created by multi-drug-resistant pathogens [13]. The antibacterial activity of titanium dioxide nanoparticles (TiO_2_NPs) arises from their capacity to generate reactive oxygen species (ROS) upon exposure to light. These ROS can infiltrate bacterial cell membranes, inducing lipid peroxidation and causing structural damage. Additionally, ROS contributes to protein and DNA damage, disrupting essential cellular functions [14].

In a specific investigation, titanium dioxide nanoparticles mediated by *Trigonella foenum-graecum* extract demonstrated remarkable antimicrobial activity against various tested Gram-positive and Gram-negative microorganisms [15]. Another study focused on *Moringa oleifera-*mediated titanium dioxide nanoparticles, revealing potent antibacterial activity against pathogens causing wound infections [16]. TiO_2_NPs synthesized through lemongrass and ginger formulation-mediated mouth paint exhibit significant antimicrobial activity against the tested oral pathogens [17]. Previous research has consistently emphasized the potential antibacterial properties of TiO_2_NPs, and our results align with these established findings by showing potent antimicrobial activity against the tested wound pathogens. The mouth paint prepared using TiO2NPs through lemongrass and ginger formulation shows less toxicity in the brine shrimp nauplii [17]. Similarly, the titanium dioxide nanoparticles exhibit a lower cytotoxic effect at lower concentrations, gradually increasing with higher concentrations.

Limitations

In our current investigations, we conducted various in-vitro analyses to assess the antimicrobial and other biomedical potential of TiO_2_NPs. Further research involving in vivo studies, including animal and clinical trials, would contribute to a more comprehensive understanding of its effects.

## Conclusions

In summary, the titanium dioxide nanoparticles produced with *Euphorbia tirucalli *displayed notable antimicrobial efficacy against the examined wound pathogens, *P. aeruginosa* and *E. coli*, as confirmed by both agar-well diffusion and time-kill curve analyses. Additionally, the synthesized nanoparticles exhibited lower cytotoxicity at both the lowest and highest concentrations. This investigation implies that environmentally friendly titanium dioxide nanoparticles could present a promising alternative in diverse therapeutic applications, offering advantages over conventional synthetic drugs with potential side effects.
